# Regulation of transcription reactivation dynamics exiting mitosis

**DOI:** 10.1371/journal.pcbi.1009354

**Published:** 2021-10-04

**Authors:** Sergio Sarnataro, Andrea Riba, Nacho Molina

**Affiliations:** Institut de Génétique et de Biologie Moléculaire et Cellulaire (IGBMC) Université de Strasbourg – CNRS – INSERM, Illkirch, France; Pázmány Péter Catholic University: Pazmany Peter Katolikus Egyetem, HUNGARY

## Abstract

Proliferating cells experience a global reduction of transcription during mitosis, yet their cell identity is maintained and regulatory information is propagated from mother to daughter cells. Mitotic bookmarking by transcription factors has been proposed as a potential mechanism to ensure the reactivation of transcription at the proper set of genes exiting mitosis. Recently, mitotic transcription and waves of transcription reactivation have been observed in synchronized populations of human hepatoma cells. However, the study did not consider that mitotic-arrested cell populations progressively desynchronize leading to measurements of gene expression on a mixture of cells at different internal cell-cycle times. Moreover, it is not well understood yet what is the precise role of mitotic bookmarking on mitotic transcription as well as on the transcription reactivation waves. Ultimately, the core gene regulatory network driving the precise transcription reactivation dynamics remains to be identified. To address these questions, we developed a mathematical model to correct for the progressive desynchronization of cells and estimate gene expression dynamics with respect to a cell-cycle pseudotime. Furthermore, we used a multiple linear regression model to infer transcription factor activity dynamics. Our analysis allows us to characterize waves of transcription factor activities exiting mitosis and predict a core gene regulatory network responsible of the transcription reactivation dynamics. Moreover, we identified more than 60 transcription factors that are highly active during mitosis and represent new candidates of mitotic bookmarking factors which could be relevant therapeutic targets to control cell proliferation.

## Author summary

Specific gene expression patterns confer particular identities to cells. During proliferation, cells undergo mitosis when chromosomes are formed and segregated into two new cells leading to a global downregulation of gene expression. Yet, cell identity is propagated from mother to daughter cells by the reactivation of gene expression at the appropriate set of genes once mitosis is completed. Mitotic bookmarking has been proposed as a mechanism to regulate this process. Indeed certain regulatory factors tag genes during mitosis to promote gene reactivation in the next cycle. Here we analyze gene expression over time measured on synchronized cell populations by using a new generation sequencing technique. To do so, we proposed a mathematical model to obtain the exact gene expression dynamics with respect to the cell-cycle progression and identified waves of genes reactivation during mitosis and the transition to the next cycle. Also, we developed a computational method that allowed us to predict key regulatory factors that drive this process and predict new candidates that could be involved in mitotic bookmarking. These regulatory factors could be relevant therapeutic targets to control cell proliferation.

## Introduction

Proliferating cells show a global downregulation of transcription during mitosis. This results from the combination of three main processes: 1) nuclear envelope breakdown leading to an increase of the volume that transcription factors (TFs) and the RNA polymerases II (RNAPII) can explore and therefore a decrease of their local concentration around gene promoters; 2) major reorganization of chromatin architecture characterized by chromosome condensation, repositioning of nucleosomes in some regulatory regions, loss of long-range interaction between enhancers and promoters and disassembling of topological associated domains (TADs); and, 3) TF-DNA binding inactivation through posttranscriptionally regulated phosphorylation. As a consequence, most TFs and the RNAPII are evicted from mitotic chromosomes and RNA synthesis is drastically reduced [1].

In spite of this global decrease of gene expression during mitosis, proliferating cells are able to maintain their cell identity and propagate regulatory transcriptional programs from mother to daughter cells [2]. Mitotic bookmarking has been proposed as a potential mechanism that could be involved in the transmission of regulatory information during the cell-cycle [3]. As initially proposed in [4], molecular bookmarking refers to transcription factors binding or histone modifications retention during mitosis that have the potential to mark specific chromatin regions and aid their reactivation in the next cycle. Indeed, a significant fraction of TFs are able to remain bound to chromatin during mitosis [5]. These mitotic-bound factors (MFs) show faster interactions with mitotic chromatin than in interphase as a reduction on binding times have been observed by single molecule tracking [5]. It is believed that non-specific chromatin or protein-protein interactions between MFs and chromosome coating proteins can explain this fast observed dynamics [5, 6]. However, it has been shown for a handful of MFs, known as bookmarking factors (BFs) [7–10], their ability to interact specifically with at least a fraction of their interphase target sites during mitosis, indicating that chromosomes are not as compacted as previously thought [10]. In fact, chromatin accessibility and nucleosomes landscape during mitosis remain unchanged on bookmarked regions bound by known BFs [11, 12]. This ability of BFs to maintain chromatin structure locally could promote a quick transcription reactivation exiting mitosis. Indeed, RNAPII occupancy exhibit a spike in promoters and enhancers during the transition between mitosis and G1 phase [13].

Transcription dynamics during mitosis and early G1 phase has recently been measured by metabolic labeling of RNA (EU-RNA-Seq) in synchronized population of Human Hepatoma cells HUH7 [14]. Remarkably, this study showed a low but detectable transcription activity during mitosis in up to 8000 genes. Furthermore, transcription reactivation occurred in intense waves exiting mitosis and early G1 phase. However, the study did not take into account that mitotic-arrested cell populations progressively desynchronized once the block was released. As a consequence, RNA measurements are performed on mixture of cells at different internal cell-cycle times. Moreover, it is not understood yet what is the precise role of mitotic bookmarking on mitotic transcription and the transcription reactivation waves. Ultimately, the core gene regulatory network driving the precise transcription reactivation dynamics remains to be identified.

In this paper we developed mathematical models and computational methods to address these open questions. First, in order to correct for the progressive desynchronization of cell populations we assumed that there is a stochastic lag time until a cell can restart the cell-cycle progression again. We characterized the distribution of lag times by analyzing how the observed fraction of mitotic cells evolves over time after the mitotic block is released. This allows us to deconvolve the EU-RNA-Seq data and produce gene expression profiles with respect to a cell-cycle pseudotime and classify the different waves of transcription reactivation in relationship with the cell-cycle progression instead of the experimental time. Moreover, we identified the key TFs determining the transcription reactivation dynamics. To do that, we developed an ISMARA-like model [15] assuming that the expression of genes at a given time point of the cell-cycle progression is a linear combination of the activities of all the TFs that can bind on their promoters. By knowing the deconvolved gene expression and integrating data on transcription factors motif affinities, we calculated the activity of every expressed TF and its role in the reactivation of transcription exiting mitosis. Indeed, this analysis allows us to divide TFs in groups according to their peak of activity with respect to the cell-cycle pseudotime and predict a core regulatory network of TFs responsible of the observed transcription waves. Interestingly, we do not see a strong correlation between known BFs as FOXA1 and the speed at which their target genes are reactivated. However, we identified around 60 TFs that are highly active during mitosis and represent new candidates of mitotic bookmarking factors. Finally, we repeat the same analysis (gene expression deconvolution and TF activity inference) using eRNA expression profiles. Interestingly, we found that, enhancer reactivation seems to occur earlier than promoter reactivation overall. Moreover, the relevant TFs for eRNA reactivation are different from the ones we detect in promoters.

## Results

### Deconvolution of gene expression data from desynchronized cell populations

**Fig 1:**
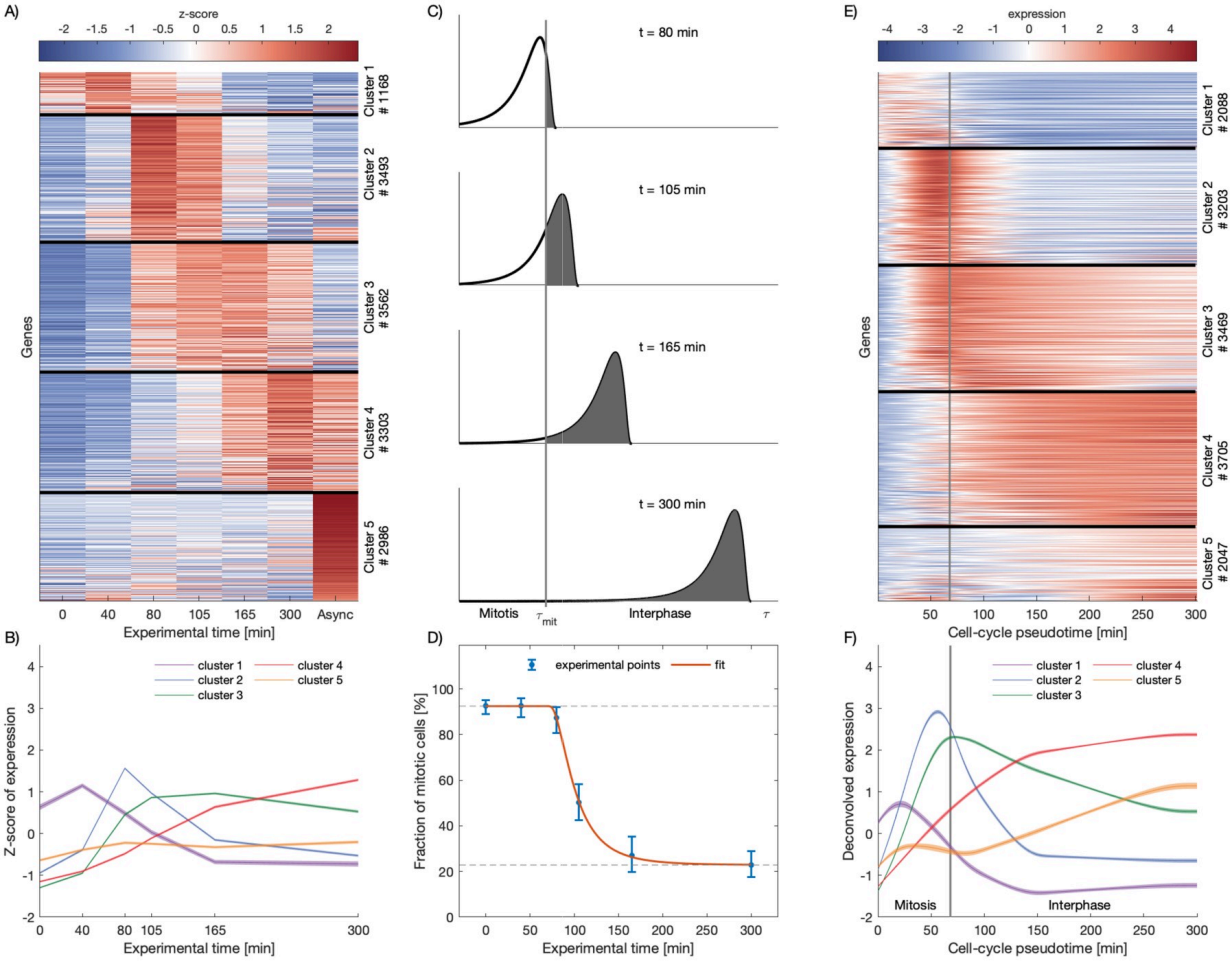
Deconvolution of gene expression data of synchronized cell populations leads to dynamic expression profiles respect to cell-cycle pseudotime. **A**: Genes can be clustered in groups according to their dynamic expression profiles over time. Each row corresponds to a gene. Black horizontal lines divide the different clusters of genes. The color scale represents the level of the Z-score of expression of each gene, as shown by the colorbar on the top. The number of genes in each cluster is indicated on the right. **B**: Average expression profiles for each cluster in A. Shades show standard errors of the mean. **C**: Distributions of cells over the cell-cycle pseudotime at different experimental time points assuming that cells have to wait a stochastic, log-normally distributed lag time to start again the cell-cycle progression after the release of nocodazole (see Methods). The gray shaded areas represent the fraction of cells that exited mitosis at different experimental times. Grey vertical indicates the time *τ*_mit_ that cells need to complete mitosis. **D**: Fraction of mitotic cells (blue dots) quantified by imaging of cells showing condensed (mitotic) and decondensed (non-mitotic) chromatin after mitotic block release (data from [14]). Error bars show confidence intervals from a simple binomial model. Red line shows the model of fraction of mitotic cells fitted to the experimental data to infer *τ*_mit_ and the parameters of the log-normal distribution, *σ* and *μ*. The dashed lines show the contamination level with asynchronous cells (top) and the contamination level with cells that did not renter the cell cycle after block was released (bottom). **E**: After the deconvolution, genes are clustered in groups according their dynamic expression profile over the cell-cycle pseudotime *τ*. The vertical white line represents *τ*_mit_ and indicates the transition between mitosis to early G1 phase. The color scale represents the level of expression of each gene, as shown by the colorbar on the top. The number of genes in each cluster is indicated on the right. **F**: Average expression profiles for each cluster in E. Shades show standard errors of the mean. Time units are in minutes.

In 2017, Palozola et al. published a study based on metabolic labeling of RNA (EU-RNA-Seq) of prometaphase synchronized population of Human Hepatoma cells (HUH7) by arresting cell-cycle progression [14] with nocodazole. EU-RNA-Seq experiments were performed to measure newly synthesized transcripts at 0, 40, 80, 105, 165 minutes after mitotic block release as well as for an asynchronous cell population. In this study, the authors highlighted the presence of low levels of transcription during mitosis and the fact that housekeeping genes and not cell-specific genes are activated earlier during the mitotic exit. We reanalyzed the EU-RNA-Seq datasets and characterized the expression dynamics at the gene level. By performing k-means clustering on the gene expression profiles, we identified 5 different groups of genes, presenting characteristic transcription reactivation dynamics over the experimental time. We successfully recapitulated the main result reported in [14] showing a time-ordered progression of transcription reactivation after block release (see Fig 1A and 1B). Our analysis, though, highlights more clearly the waves of gene reactivation as every group of genes reach the maximum of transcription at a specific time followed by a decrease of activity (see Fig 1A and 1D).

Notably, using cell imaging, Palozola et al. measured how the fraction of mitotic cells evolves over time once the mitotic block is released (see Fig 1D and [14]). This indicates that mitotic-arrested cell populations progressively desynchronize after washing out nocodazole and therefore the EU-RNA-seq experiments were performed on mixtures of cells at different internal cell-cycle times. In addition, at every experimental time point, there was contamination from asynchronous cells that escape mitotic block. To address these issues, we developed a mathematical model that is able to correct for the desynchronization and the contamination with asynchronous cells. To do so, we assumed that after mitotic block release there is a stochastic lag time until cells can start again the cell-cycle progression that, for simplicity, is log-normal distributed with a certain mean *μ* and standard deviation *σ*. We introduced the concept of *internal cell-cycle pseudotime* *τ*, defined as the effective cell-cycle time progression of a cell, starting once the lag time is over. We then assumed that there is an average time *τ*_mit_ that cells need to complete mitosis and that, within the maximum experimental time (6h), cells do not reenter mitosis. All this, allowed us to obtain a probability function that describes the distribution of cell-cycle pseudotimes of cells within the cell population given a certain experimental time *t* since the mitotic-block release (see Fig 1C and Methods for a detailed mathematical derivation). Notice that this probability distribution allows us to calculate the expected fraction of mitotic cells and its evolution over time (white areas in Fig 1C) which can be used to estimate the parameters *τ*_mit_, *μ* and *σ* by fitting the theoretical function to the cell imaging data (see Fig 1D and Methods). This led to an estimated median lag time of 30 minutes and an average time to complete mitosis of 67 minutes.

Next, we assumed that the time-dependent EU-RNA-Seq data measured at a certain experimental time is basically the population average over the expressions of individual cells at different cell-cycle pseudotimes. Then, the probability distribution of cell-cycle pseudotime fitted before can be used to deconvolve the experimental data and obtain as a result the expression of genes with respect to the cell-cycle pseudotime *τ* (see Methods and S1 Table). Importantly, our method allowed us to uncover the gene expression dynamics during the cell-cycle progression and highlight the transition between mitosis and early G1 phase as well as report the peak of expression in relationship with this transition. As can bee seen for some selected genes in Fig B in S1 Appendix, our deconvolution approach produces sharper dynamical waves and a general shift towards smaller times indicating that reactivation tends to be more intense and earlier than measured experimentally. These dynamical features are somehow hidden in the raw experimental data due to the natural desynchronization of cell populations. Overall, we identified 5 different clusters of genes showing distinct transcription reactivation dynamics over the cell-cycle pseudotime *τ*: early-mitotic, late-mitotic, mitotic-G1 and early-G1 waves (see Fig 1E and 1F). Notice that, although the clusters look similar before and after the deconvolution, thanks to our analysis, we are able to identify genes that are reactivated during mitosis and before cytokinesis. Indeed, a cluster around 2000 genes showed an expression wave very early during mitosis, presumably around metaphase, while a large fraction of genes reach their reactivation peak just before exiting mitosis, during telophase or during the transition to early G1 phase, as shown in Fig 1E and 1F. In summary, our analysis allows us to correct for desynchronization of cell populations and study gene expression dynamics with respect to the cell-cycle pseudotime highlighting the waves of transcription in relationship with the transition between mitosis and interphase.

### Transcription factor activity dynamics during mitosis and early G1 phase

**Fig 2:**
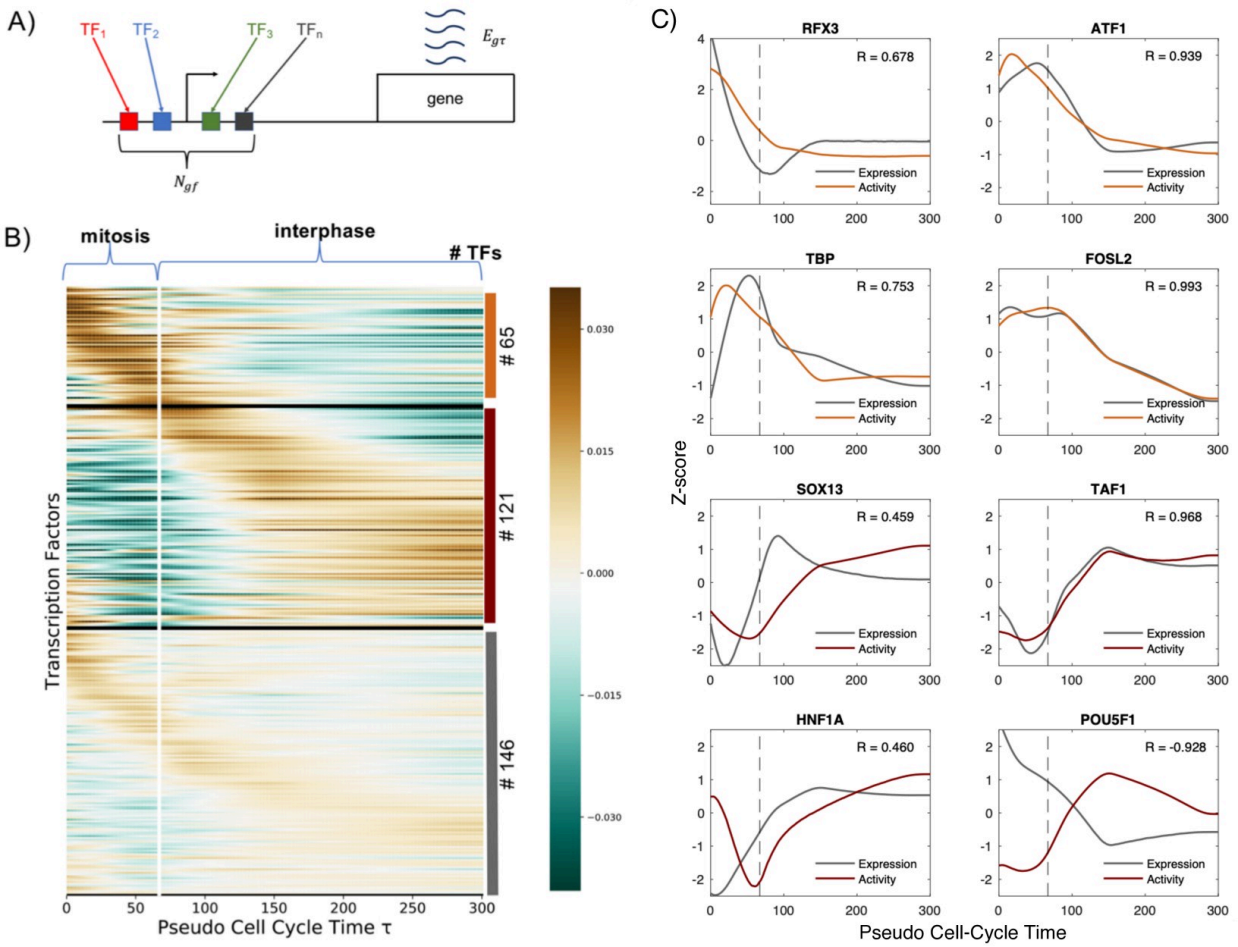
Transcription factor activity dynamics during mitosis and early G1 phase. **A**: Schematic representation of the model: the expression *e*_*gτ*_ of the gene *g* at cell-cycle pseudotime *τ* is a linear combination of the activities of different transcription factors *f* binding the promoter of *g*. *N*_*gf*_ represents the entries of a matrix **N** containing the number of sites for TF *f* associated with promoter of the gene *g*, taking into account the affinity between the motif of *f* and the sequence of the promoter. **B**: Heatmap showing TF activities for expressed TFs in HUH7. The TF activity level is color coded and the scale is shown in the colorbar. The vertical white line represents *τ*_mit_, and indicates the transition between mitosis and interphase. TFs are clustered using k-means according to their activity dynamics over the cell-cycle pseudotime *τ*. Three main groups are detected: mitotic-active, earl-G-active and steady-active. Within each cluster TFs are sorted according to when their maximum activity occurs. On the right, the number of TFs belonging to each cluster is indicated. **C**: Activities of mitotic-active (orange curves) and early-G1-active (red curves) TFs that show a high amplitude dynamics. Grey lines show the gene expression dynamics of the corresponding TF genes. Z-score transformation was applied to facilitate the comparison of the expression and activity dynamics. Pearson correlation coefficients between the TF activities and expressions are shown in each panel. Dashed lines represent *τ*_mit_. Time units are minutes in all plots.

The transcription waves identified in the previous section are driven most likely by regulatory transcriptional programs that results from the interplay between transcription factors (TFs) and epigenetic elements such as DNA methylation and chromatin modifications. Here, we will focus on analyzing which TFs may be the principal drivers of the transcription reactivation dynamics. To do so, we implemented the ISMARA method [15], a simple model that relates gene expression with TFs activities. Briefly, we assumed that the normalized log-transformed expression *e*_*gτ*_ of a gene *g* at cell-cycle pseudotime *τ* can be obtained as a linear combination of the cell-cycle dependent activities *A*_*fτ*_ of all TFs *f* that can potentially regulate the gene (see Fig 2A). The model can be summarized by the following equation:

$$\begin{array}{c} e_{g\tau }=\sum_{f}N_{gf}A_{f\tau } \end{array}$$ (1)

where the values *N*_*gf*_ represent the entries of a matrix **N** containing the number of binding sites for the TF *f* associated with the gene promoter *g*. From the analysis we excluded TFs that are not expressed in HUH7. To build the matrix **N** we used computational predictions of TF binding sites (TFBSs) from SwissRegulon [16]. This database contains TFBS predictions on human gene promoters using MotEvo [17], a probabilistic Bayesian method that combines known sequence-specificities of TFs and sequence conservation. Notice, that with this model we omit long-range regulatory inputs that may be mediated by TF binding in enhancers. However, to determine reliably which enhancer-promoter interactions are functional is not an straightforward task and, to the extent of our knowledge, there is no adequate dataset in our system that could inform us. Moreover, it is known that many of the enhancer-promoter interactions are disabled during mitosis and reformed dynamically during the transition to the G1 phase [18]. On the other hand, assuming that TFs act in a linear manner on promoters is a strong oversimplification. Quantitative analysis on single promoters have shown that non-linearities and cooperatively between TFs are important features of gene regulation [19]. However, our aim, by fitting a linear model to the gene expression data, is not to have a precise mechanistic model able to reproduce expression profiles but to robustly infer TFs that are potential drivers of transcription reactivation exiting mitosis. Notice that more complex models involving larger number of unknown parameters could in principle be used, however, the risk of overfitting increases dangerously. As shown previously, this linear model based on regulatory interactions in promoters may not explain with a high accuracy the expression patterns at the single gene level but is an adequate model to robustly infer TF activities combining the information from many genes in a simple but rigorous statistical framework [15]. Finally, to avoid overfitting we introduced a regularization term that enforces smooth TF activities over the cell-cycle pseudotime and we calibrated its strength using a cross-validation approach (see Methods for further mathematical details).

Our analysis allows us to infer the activity of 332 TFs (see S2 Table). This can be understood as a dimensionality reduction approach as we describe the problem of transcription reactivation with much fewer parameters, since we pass from the analysis of thousands of genes to only hundreds of TFs. To analyze the activity dynamics we use k-means and divide them in 3 clusters, according to their profile over *τ* that we named: mitotic-active, early-G1 active and steady. We showed that almost 19% of TFs present positive activity during mitosis, with a peak in the first minutes, and then progressive decrease of activity. Conversely, 36% of TFs present a negative activity during mitosis, and then a high activity in early G1. Lastly, the remaining 45% of TFs show a moderate amplitude in their dynamics suggesting that they play a minor role on transcription reactivation dynamics (the results are shown in Fig 2B and average activities in each cluster in Fig D in S1 Appendix). Among TFs that are active during mitosis, we obtain known bookmarking factors as C/EBP, HSF1, TBP, GATA1 and ESRR*β* [3, 14, 20–22] reassuring that our approach is able to identify relevant TFs. Remarkably, among the TFs for which we predict the highest mitotic activity are ATF1, MYBL2 and MYB which are well known master regulators of the cell cycle. This may indicate a potentially interesting link between mitotic bookmarking and cell cycle regulation. Moreover, activities of TFs that are annotated to the Gene Ontology category cell-cycle show a dynamics during mitosis and early G1 phase with a similar amplitude as the mitotic-active and early-G1 active clusters (see Fig E in S1 Appendix). Interestingly, by sorting TFs according to when their highest peak of activity occurs, we observed waves of activity suggesting an intrinsic TF ordering with respect to their role on the temporal reactivation of transcription after mitosis (see Fig 2B).

Determining the molecular mechanisms underlying the TF activity dynamics that we inferred goes beyond the scope of this study. However, we can have a first hint by analyzing the cross-correlation between the TF activity and the expression of the corresponding TF gene. Indeed, a strong correlation indicates that changes in TF expression may be responsible for changes in activity. Furthermore, a lag time of the TF activity with respect to its expression reflects possible delays on the accumulation of active protein due to mRNA and protein half-lives or post-transcriptional regulation. In addition, positive or negative correlation reveals that the TF acts mainly as an activator or repressor respectively. On the contrary, absence of correlation could be explained by other processes that may influence TF activity as post-trancriptional modifications, cellular localization or the interaction with other co-factors [15]. Thus, we computed the maximum cross-correlation coefficients and the lag times between the expression and the activity of all TFs. We obtained a bimodal distribution of maximum correlation coefficients with more extreme modes than the ones obtained with random data (see Fig F in S1 Appendix). Interestingly, this result can be used to identify activators and repressors (53% and 47% of all TFs, respectively). As examples, in Fig 2C, we show activities of mitotic- and early-G1-active TFs with the highest amplitude dynamics together with their expression profiles. Interestingly, TBP, TAF1 and FOSL2 show a high positive correlation indicating that they act mainly as activators and their activities may be regulated at the transcriptional level. On the other hand, SOX13 and HNF1A show a clear lag time between expression and activity which could reflect the delay on the accumulation of active protein due to mRNA and protein half-lives or post-transcriptional regulation. Notably, POU5F1 shows a strong negative correlation which suggests that acts mainly as a repressor as it has been suggested previously [23]. In summary, our analysis not only allows us to identify the activity dynamics of key TFs involved in transcription reactivation but also provides preliminary hints on the molecular mechanisms that may be involved in such dynamics.

### Identification of the Core Regulatory Network responsible for the transcription reactivation after mitotic exit

Next, we wanted to identify the TFs, among the 332 for which we are able to infer activities, that have a major role on the reactivation of transcription exiting mitosis. Namely, the key TFs that if perturbed may affect more significantly the measured gene expression patterns. To do so, we calculated the fraction of explained variance as a measure of the performance of our model to fit the data. Then, we defined a TF importance score as the reduction on fraction of explained variance when the TFs is removed as an explanatory variable from the multiple linear regression model (see Methods for further details). A list of all TFs sorted according to their importance score is given in the S3 Table.

**Fig 3:**
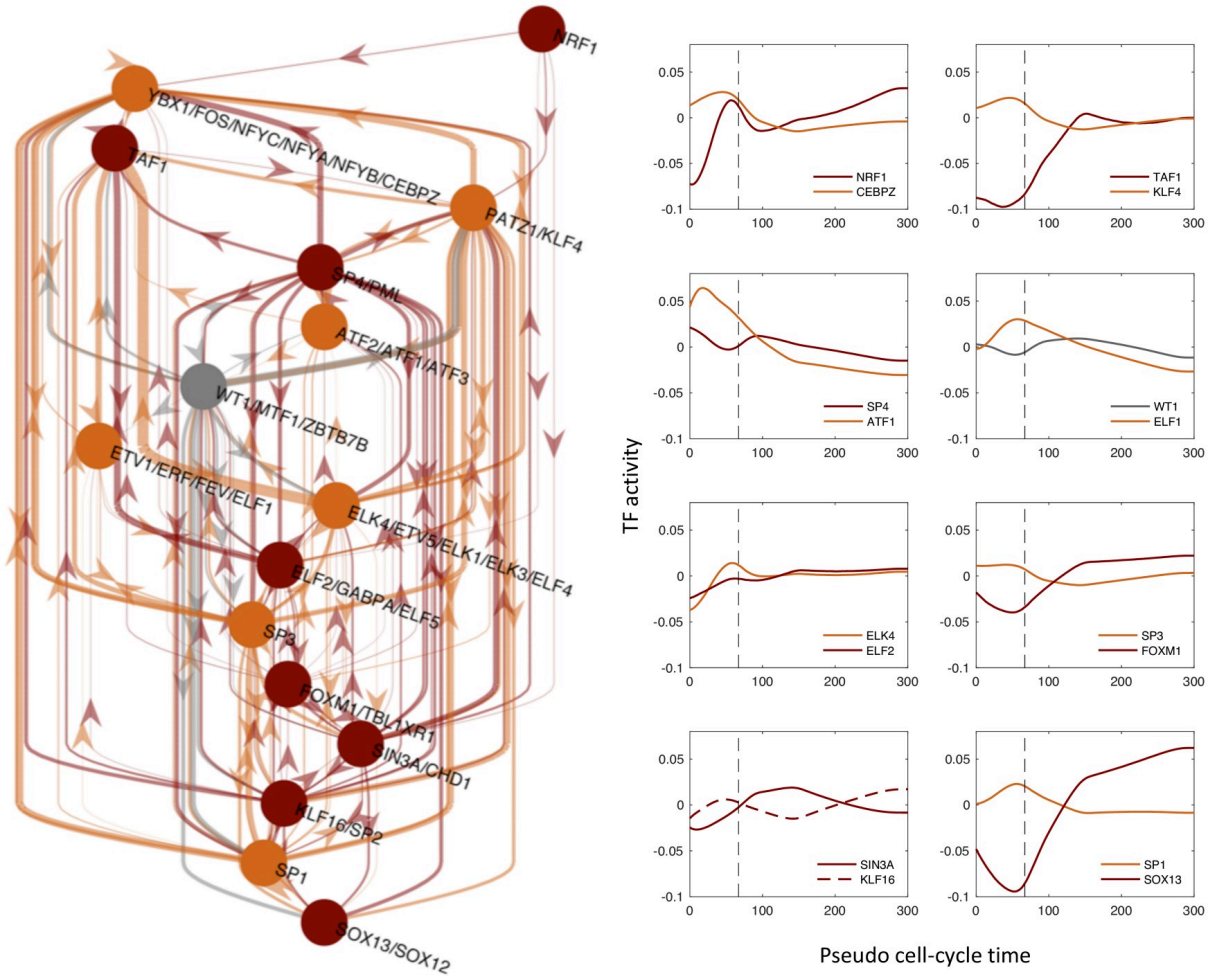
Identification of the Core Regulatory Network responsible for the transcription reactivation after mitotic exit. Left: Inferred core regulatory network (CRN) by selecting the top 5% of the TFs according to their importance in explaining the gene expression patterns and their inferred time-dependent activities. Colours indicate the cluster to which TFs belong, in accordance with the Fig 2B. Right: curves show the TF activities of the network nodes. Note that the majority of TF motifs are associated uniquely to a single TF, while other motifs are shared by more than one TF leading to the same TF activity for all TFs that share the same motif. The time units are in minutes.

Furthermore, in Fig 3 we show a Core Regulatory Network (CRN) where the nodes are formed by the 5% top most important TFs (see Fig G in S1 Appendix) and the links represent potential regulatory interaction between the selected TFs according to the presence of TF binding sites in their promoters, i.e. the non-zero entries of the matrix *N*_*gf*_ (see Methods). Interestingly, the CRN shows a large number of regulatory links (149 connections) while networks with the same number of TFs randomly chosen contain a smaller number of connections based on the matrix *N*_*gf*_ (36 on average). Thus, the high interconnectivity of our CRN suggests that the identified TFs may be related functionally. Indeed, some of them have been reported to be involved in cell-cycle or cell proliferation and growth such as ATF1, FOS, CEBPZ, SP3 and KLF4 [24]. Moreover, the CRN structure shows multiple feedback loops rather than a hierarchical network as one could expect taking into account the observed sequential waves of transcription reactivation. This type of structure has the potential to show cycling dynamics which may be important not only for the reactivation after mitosis but for the regulation of transcription across the whole cell-cycle. In conclusion, these TFs could represent relevant therapeutic targets to control cell proliferation.

### Bookmarking and transcription reactivation kinetics

**Fig 4:**
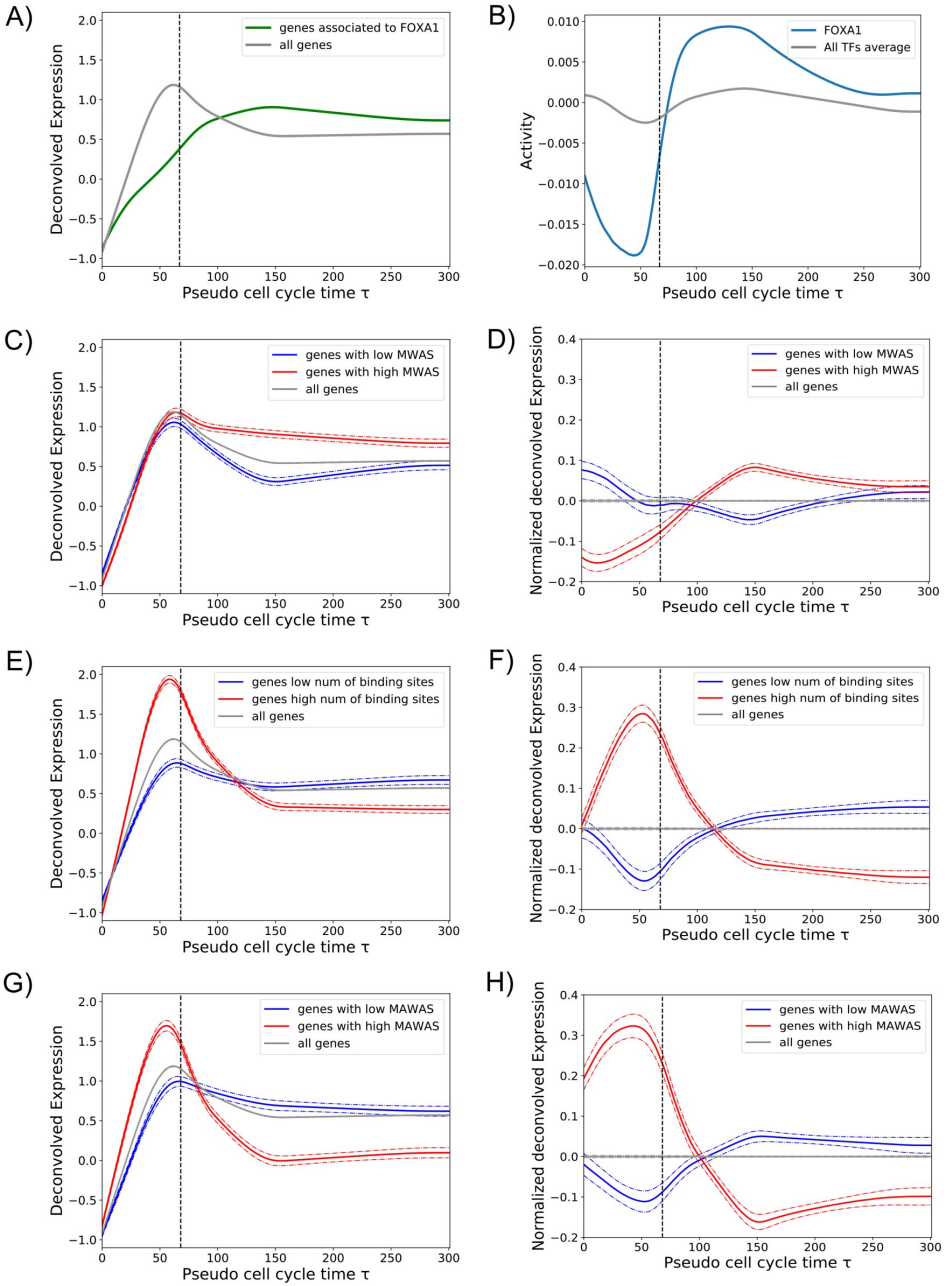
Bookmarking and transcription reactivation dynamics. **A**: The average expression of all genes (grey line) was compared with the average expression of FOXA1 target genes during mitosis (green line). **B**: The activity of FOXA1 (blue line) in comparison with the average activity of all TFs (grey line). **C**: Gene expression pattern as a function of the promoter MBS (mitotic binding score). The average expression of all genes (grey line) was compared to the average expression of genes whose promoters tend to be regulated by TFs with high MBF (red line) and low MBF (blue line) (see Methods). **D**: The same as in panel C, but gene expression patterns have been processed as described in Methods, Eq 16. **E**: Gene expression pattern as a function of the total number of TF binding sites in the promoter. The average expression of genes with large (red) and low (blue) number of promoter binding sites are compared to the overall average expression (grey line). **F**: The same as in panel E, but gene expression patterns have been processed as described in Methods, Eq 16. **G**: Gene expression pattern as a function of the promoter MAS (mitotic activity score, see Methods). The average expression of genes with high (red) and low (blue) MAS are compared to the overall average expression (grey line). **H**: The same as in panel G, but gene expression patterns have been processed as described in Methods, Eq 16. Dashed vertical lines in all panels indicate *τ*_mit_.

Next, we investigated the role of mitotic bookmarking in the transcription reactivation dynamics. To do that, we analyzed the expression of genes associated to FOXA1, a liver-specific factor and one of the first identified bookmarking factors. We used mitotic ChIP-Seq data from a study of Caravaca et al. [7]. We selected the genes associated to FOXA1 ChIP-Seq peaks (see Methods) and we calculated the average expression and compared it with the overall average gene expression. Interestingly, genes associated to FOXA1 reach their activation peak later than the overall peak of gene expression that occurs during the transition between mitosis and early G1 phase (see Fig 4A). This is expected as FOXA1 regulates mainly liver-specific genes and, as shown by Palozola et al., these genes are reactivated later than genes related to basic cell functions [1]. Then, we compared the activity of FOXA1 with the average activity of all TFs, revealing a valley in activity during mitosis (see Fig 4B), in accordance with the results shown in Fig 4A. Compared to the other TFs during mitosis, FOXA1 belongs to the 25 percentile of factors with largest negative activity and in the 12 percentile of the smallest average activity during mitosis. These results suggest that FOXA1, despite its presence on mitotic chromosomes through specific and non-specific interactions, is not sufficient to promote quick transcription reactivation. However, we cannot exclude that mitotic binding of FOXA1 may be functional. For example, it may play a structural role by keeping the chromatin open to promote binding of other TFs.

To scale up this analysis we took advantage of a recent large scale study by Raccaud et al. in 2019. The authors were able to systematically measure the mitotic chromosome binding of 501 TFs in mouse fibroblast cells by live-imaging cell lines carrying exogenous fluorescence constructs. The mitotic bound fraction (MBF) was defined as the fraction of fluorescence signal located on mitotic chromosomes over the total cell signal. According to this score the TFs were divided in three categories (enriched, intermediate and depleted) indicating their capacity to bind mitotic chromosomes and their potential to be bookmarking factors. We then assumed that human TFs in HUH7 cells behave similar as their mouse paralogs and assigned the corresponding MBF score. We hypothesized that genes regulated by TFs with high MBF should be ready to be reactivated earlier. To test this, we calculated a Mitotic Binding Score (MBS) for each promoter as the weighted average MBF of the TFs that regulate a given gene promoter weighted by the number of their binding sites. Then, we divided genes in high and low MBS and calculated the average expression of these two groups (see Methods for further information). Genes associated to high MBS, i.e gene that tend to be regulated by TFs with high MBF, did not show a faster reactivation dynamics but a significant larger expression during early G1 phase (see Fig 4C and 4D). Consistently, we showed no significant difference between the MBF distribution of TFs with high activity during mitosis or during early G1 (Fig H in S1 Appendix). These results indicate that there is an absence of correlation between TF mitotic binding and TF mitotic activity and quick transcription reactivation of their target genes. We cannot rule out that the absence of correlation could be due to the fact that MBF scores were measured with an artificial system in a different cell line of a different organism. However, the absence of correlation may indicate that mitotic bound factors, as in the case of FOXA1, may have a structural function by keeping chromatin open during mitosis and the speed of transcription reactivation may be then regulated by other determinants.

Next, we studied whether promoter architecture could be one of the determinants of early transcription reactivation. Surprisingly, just the total number of binding sites within the gene promoter is a strong feature to predict early or late reactivation. Indeed, average expression of genes with large number of binding sites shows a quick transcription reactivation during mitosis, in contrast to a reactivation during early G1 of genes with small number of binding sites (see Fig 4E and 4F). Two non-exclusive mechanisms could explain why strong promoters reactivate earlier: first, gene promoters with more TF binding sites may be easier to keep accessible during mitosis as more TFs could compete against nucleosomes leading to nucleosome free regions. Second, large number of binding sites may facilitate TFs to find the promoters, increasing the chances to recruit the transcriptional machinery. Finally, as expected, genes that have a large number of binding sites for TFs with a high inferred activity during mitosis (high Mitotic Activity Score, MAS), showed a high mitotic transcription and a quick transcription reactivation (see Fig 4G and 4H, and S4 Table). Overall, we believe that our method allows to identify new bookmarking factors that should not only bind mitotic chromosomes but be able to bind specific DNA binding sites during mitosis. In addition, we predict that promoters with large number of binding sites for these TFs should show a higher degree of chromatin accessibility during mitosis.

### Deconvolution of eRNA expression and inference of TF activities on enhancers

**Fig 5:**
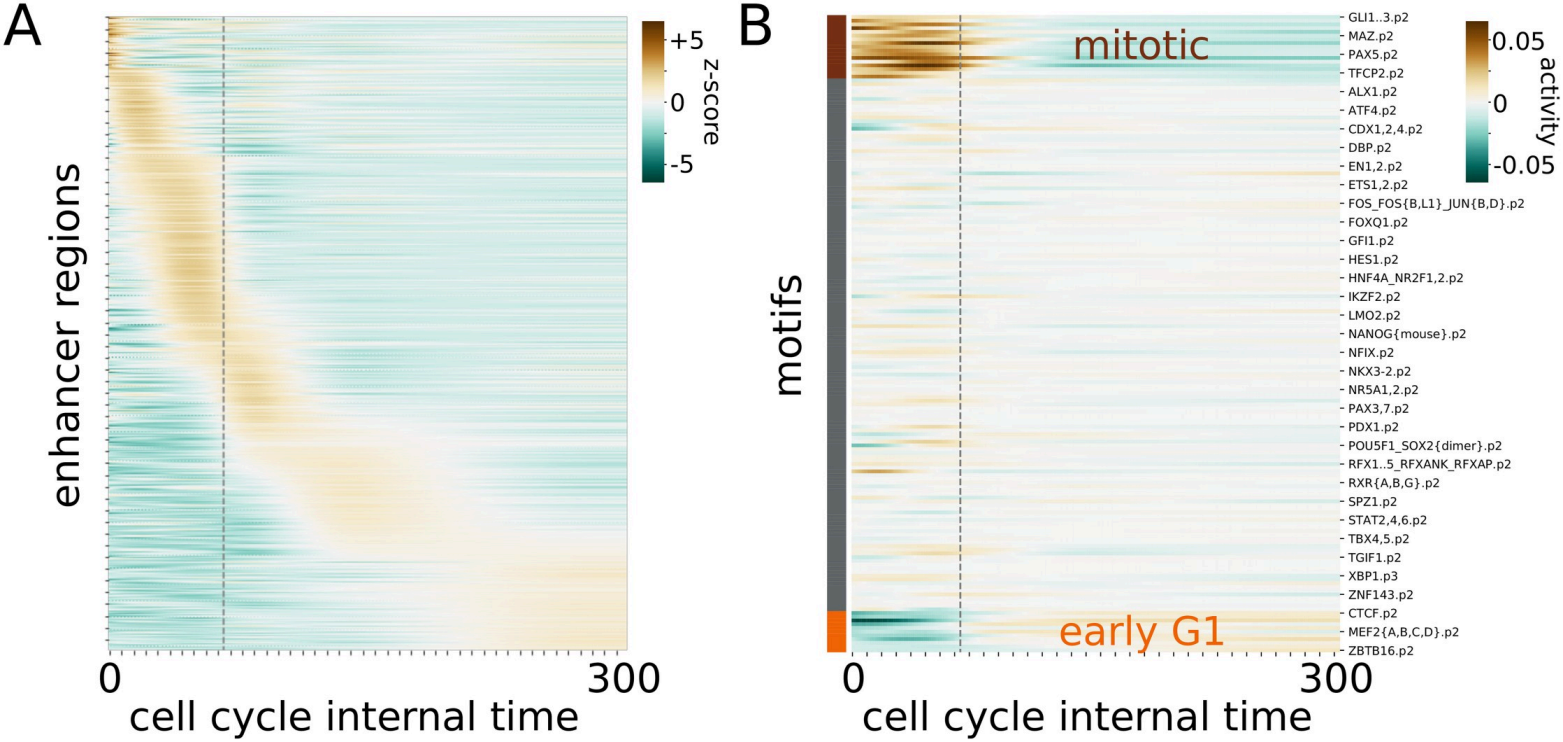
Reactivation of eRNAs and TF activities on enhancers. **A**: Deconvolved expressions (z-score) of the enhancer regions across the cell-cycle pseudotime. The original expression values have been obtained from Palozola et al. and applied the same deconvolution approach as with promoters (see Fig 1 and Methods). **B**: Activity of TFs inferred from the deconvolved eRNA expression profiles in A. TFs can be clustered in three groups based on their activities. The first group is active before mitosis (brown), the second one has a minor influence on the mitotic activity and reactivation process (gray), and the latter (orange) is active at the beginning of a new cell cycle. Time units are in minutes.

In their original study Palozola et al. suggested that the reactivation of genes and enhancer RNAs is concomitant [14]. Therefore, after analyzing the effect of TFs on promoter regions, we wondered which TFs play a role in the regulation of enhancers activity during mitosis exit. To do so, we analyzed the eRNA expression profiles obtained from the EU-RNA-seq experiments on synchronized cell populations analyzed before. First, we deconvoved the eRNA expression using the same method as with genes to correct for cell desynchronization. Examples of single deconvoled eRNAs together with the raw experimental data are shown in Fig I in S1 Appendix. Deconvolved eRNA expression show also progressive waves of reactivation with respect to the cell-cycle pseudotime (Fig 5A). Interestingly, after the deconvolution, eRNA expression shows overall a earlier reactivation than genes. Indeed, 52% of the enhancers analyzed show a peak in expression before cytokinesis compared to 41% of the genes.

The transcription factors responsible for the trend in enhancer expressions can be identified with the ISMARA model on the motif predicted within the enhancer regions. Among the top 20 motifs identified from the promoter analysis and the enhancer analysis, only two are in common: CEBPA and FOXC1. CEBPA is known to interact with CDK2,4 and to be crucial in embryonic growth [25]. FOXC1 inhibits cell growth and stops cells in G1 [26]. Overall, little overlap between the two lists of motifs is consistent with the current model that the subset of TFs that bind specifically to promoters or enhancers are different [27]. Finally, the TF activities can be clustered into three groups, the first active before mitosis, the second with little change in activity and the last one where the factors increase their activity right after mitosis (Fig 5B).

## Discussion

Cell identity maintenance in proliferating cells is a biological process that has crucial implications in developmental biology, regenerative medicine and cancer. Nevertheless, the precise molecular mechanisms responsible for the transmission of regulatory information from mother to daughter cells are not fully understood yet. Epigenetic mechanisms related to DNA meyhylation and chromatin modifications have been shown to play an important role [28, 29]. Recently, mitotic bookmarking by TFs through specific DNA binding on mitotic chromosomes has been proposed as a mechanism to reinforce cell identity maintenance during cell division. In this paper we studied the regulation of transcription reactivation exiting mitosis and the connection with mitotic bookmarking.

First, we reanalyzed time-dependent EU-RNA-seq data on synchronized cell populations by a mitotic arrest, to correct for the progressive desynchronization of cells after block release. This allowed us to estimate sharper gene expression profiles with respect to a cell cycle pseudotime that can be precisely placed with respect to an explicitly defined transition between mitosis and early G1 phase. As opposed to the results reported by Palozola et al [14], we are able to predict which genes are reactivated early in mitosis and before or after cytokinesis. Remarkably, we identified a set of genes that show a very early wave of transcription reactivation during mitosis. However, the majority of genes showed a peak of transcription at telophase or during the transition between mitosis and G1.

Next, we estimated TF activity dynamics of 332 expressed TFs by fitting a multiple linear model to the deconvolved gene expression profiles. We observed time-dependent waves of TF activities suggesting an intrinsic TF ordering with respect to their role on transcription reactivation after mitosis. In addition, we investigated whether TFs previously reported to bind mitotic chromosomes were responsible for a faster reactivation dynamics. Surprisingly, we did not find a strong correlation between genes regulated by mitotic bound TFs and the speed of reactivation. However, our approach allowed us to identify around 60 TFs that are highly active during mitosis and represent new candidates of mitotic bookmarking factors of promoters. In addition, we found enhancer-specific TFs responsible for eRNA reactivation. As opposed to the comprehensive study of Raccaud et al. [5] where mitotic binding was reported by microscopy, our analysis relay on the potential impact of TFs on mitotic gene expression. Therefore, we hypothesize that the interactions of these predicted bookmarking factors with their specific target sites during mitosis may be the molecular mechanisms responsible for mitotic transcription and/or transcription reactivation. Moreover, we hypothesize that these specific interactions may also play an important role maintaining chromatin accessibility on mitotic chromosomes. Further experimental work would be needed to validate our hypothesis and predictions.

Moreover, we reconstructed a core regulatory network underlying the dynamics of transcription reactivation exiting mitosis, by selecting the key TFs that showed the highest explanatory power in our multiple linear regression model. Then, we propose a list of candidates to be crucial players, in association with other epigenetic factors, in the process of reactivating the gene expression during mitotic exit and the first stages of interphase, ensuring the maintenance of cell identity. We predict that these TFs could represent relevant therapeutic targets to control cell proliferation. Further experiments are required to validate our predictions and prove the active role of TFs on chromatin accessibility and 3D structure.

## Methods

### Fitting of model parameters for deconvolution of gene expression data

To estimate gene expression dynamics with respect to an internal cell-cycle pseudotime, we assumed that after the release of the synchronization there is a stochastic lag time until cells can start again the cell-cycle progression. According to our model, the cell-cycle progression of a cell is represented by the internal cell-cycle pseudotime *τ* = *t* − *η*, where *t* is the experimental time and *η* is the stochastic lag time that the cell had to wait until the cell-cycle progression was restarted again. We further assumed that the lag time *η* is log-normally distributed with a certain mean *μ* and standard deviation *σ*. Then, the probability of finding a cell in the population with an internal cell-cycle pseudotime *τ* at a given experimental time *t* can be written as:

$$\begin{array}{c} P\left(\tau |t\right)d\tau =\frac{1}{\sqrt{2\pi \sigma^{2}}\left(t-\tau \right)}e^{-\frac{{\left(\text{log}\left(t-\tau \right)-\mu \right)}^{2}}{2\sigma^{2}}}d\tau \end{array}$$ (2)

Assuming that cells require an average time *τ*_mit_ to complete mitosis we can calculate the fraction of cells waiting for mitosis to be finished as $q\left(t\right)=\int_{0}^{\tau_{\text{mit}}}P(\tau |t)d\tau$ and solving the integral we obtain:

$$\begin{array}{c} q\left(t\right)=\{\begin{array}{cc} \frac{1}{2}-\frac{1}{2}\text{erf}\left(\frac{\text{log}\left(t-\tau_{\text{mit}}\right)-\mu }{\sqrt{2}\sigma }\right) & t>\tau_{\text{mit}}\\ 1 & t\leq \tau_{\text{mit}} \end{array} \end{array}$$ (3)

Then, we fit the parameters of the stochastic model (*τ*_mit_, *μ* and *σ*) by using data from [14] on the time evolution of the number of mitotic cells observed after synchronization treatment release. To do so, we define the likelihood of the data based on the assumption that the cell counts follow a binomial distribution with probability *q*(*t*). Thus,

$$\begin{array}{c} L=\prod_{i}q{\left(t_{i}\right)}^{n_{i}^{\text{mit}}}{\left(1-q\left(t_{i}\right)\right)}^{n_{i}^{\text{tot}}-n_{i}^{\text{mit}}} \end{array}$$ (4)

where $n_{\text{tot}}^{i}$ and $n_{\text{mit}}^{i}$ are, respectively, the total number of cells and the number of cells in mitosis counted at experimental time *t*_0_ = 0 minutes, *t*_1_ = 40 minutes, *t*_2_ = 80 minutes, *t*_3_ = 105 minutes, *t*_4_ = 165 minutes and *t*_5_ = 300 minutes. Then, the log likelihood can be written as:

$$\begin{array}{c} \text{log}L=\sum_{i}n_{i}^{\text{mit}}\;\text{log}\;\left(q\left(t_{i}\right)\right)+\sum_{i}\left(n_{i}^{\text{tot}}-n_{i}^{\text{mit}}\right)\;\text{log}\;\left(1-q\left(t_{i}\right)\right) \end{array}$$ (5)

By performing an optimization of Eq 5 (python *scipy optimize* package, *Nelder-Mead* algorithm), we can infer the parameters *τ*_mit_, *μ* and *σ*, obtaining, respectively, 67min ± 8, 3.4 ± 0.4 and 0.7 ± 0.3. Once the parameters have been inferred, the probability *P*(*τ*|*t*) is fully determined and, therefore, we can recover the gene expression with respect to the internal cell-cycle pseudotime *τ* using the following convolution equation:

$$\begin{array}{c} r_{g}\left(t\right)=\int_{0}^{t}E_{g}\left(\tau \right)P\left(\tau |t\right)d\tau \end{array}$$ (6)

where *r*_*g*_(*t*) represents the expression of the gene *g* at experimental time *t* (given by the EU-RNA-Seq data), *E*_*g*_(*τ*) is the expression of the same gene *g* at the cell-cycle pseudotime *τ*. This equation basically reflects that the gene expression measured at a certain experimental time is the population average over the expressions of cells at different cell-cycle pseudotimes. In case of perfect synchronization over time, the probability *P*(*τ*|*t*) would become a Dirac delta function and the gene expression in both times would be the same. Furthermore, we took into account that the samples were contaminated by a fraction *π*_*M*_ = 0.23 of cells that never exited mitosis and a fraction *π*_*I*_ = 0.075 of cells that did not respond to the mitotic block and stayed in interphase as shown by the cell imaging data (see Fig 1D and [14]). It means that only a fraction *π*_*C*_ = 1 − *π*_*M*_ − *π*_*I*_ starts again the cell cycle progression within the duration of the experiment. Then, this can be summarized by describing the measured gene expression as a mixture of the three cell populations as follows:

$$\begin{array}{c} r_{g}\left(t\right)=\pi_{C}\int_{0}^{t}E_{g}\left(\tau \right)P\left(\tau |t\right)d\tau +\pi_{M}E_{g}\left(0\right)+\pi_{I}E_{g}^{I} \end{array}$$ (7)

$$\begin{array}{c} r_{g}^{a}=f_{\text{mit}}\int_{0}^{\tau_{\text{mit}}}E_{g}\left(\tau \right)d\tau /\tau_{\text{mit}}+f_{I}E_{g}^{I} \end{array}$$ (8)

where, $E_{g}^{I}$ is the average expression during interphase and an extra equation is included to relate the gene expression $r_{g}^{a}$ measured on an asynchronous cell population as a weighted average of the gene expression during mitosis and during interphase where the weights reflect the fraction of the cell-cycle duration *T*_*C*_ that cells expend on average in each phase, i.e *f*_mit_ = *τ*_mit_/*T*_*C*_ and *f*_*I*_ = 1 − *f*_mit_.

Then, to perform the deconvolution we discretized the cell-cycle pseudotime into small intervals (*δτ* = 1 min) and expressed the Eqs 7 and 8 into matricial from: **r**_*g*_ = *M***E**_*g*_, where the expression vectors are defined as $r_{g}=\left(r_{g}\left(0\right),r_{g}\left(t_{0}\right),r_{g}\left(t_{1}\right),\ldots ,r_{g}^{a}\right)$ and $E_{g}=\left(E_{g}\left(0\right),E_{g}\left(\delta \tau \right),E_{g}\left(2\delta \tau \right),\ldots ,E_{g}^{I}\right)$ and the matrix *M* is the sum of three components: *M* = *M*_*C*_ + *M*_*M*_ + *M*_*I*_ that account for the three distinct cell populations. First, the cell-cycle matrix *M*_*C*_ models the desynchronization of the cells that re-enter the cell cycle, as in Eq 6, and can be written as:

$$\begin{array}{c} M_{C}=(\begin{array}{cc} \pi_{C}P & \begin{array}{c} 0\\ 0\\ \cdots \\ 0 \end{array}\\ \begin{array}{cccc} 0 & 0 & \cdots & 0 \end{array} & 0 \end{array}) \end{array}$$ (9)

where *P* is the discrete version of Eq 2. Second, the mitotic matrix *M*_*M*_ adds to the model the contribution of the cells that are still in mitosis by mapping them into *τ* = 0. Its explicit form is:

$$\begin{array}{c} M_{M}=\pi_{M}(\begin{array}{cc} \left(\begin{array}{ccccccc} 1 & 0 & \cdots & 0 & \cdots & 0 & \\ 1 & 0 & \cdots & 0 & \cdots & 0\\ \cdots & \cdots & \cdots & \cdots & \cdots & \cdots \\ 1 & 0 & \cdots & 0 & \cdots & 0 \end{array}\right) & \begin{array}{c} 0\\ 0\\ \cdots \\ 0 \end{array}\\ \begin{array}{ccccccc} 0 & 0 & 0 & \cdots & \cdots & \cdots & 0 \end{array} & 0 \end{array}) \end{array}$$ (10)

And third, the interphase matrix *M*_*I*_ exploits the asynchronous dataset to infer the average expression levels during interphase. The matrix takes the following form:

$$\begin{array}{c} M_{I}=(\begin{array}{cc} \left(\begin{array}{ccccccc} 0 & 0 & \cdots & 0 & \cdots & 0 & \\ 0 & 0 & \cdots & 0 & \cdots & 0\\ \cdots & \cdots & \cdots & \cdots & \cdots & \cdots \\ 0 & 0 & \cdots & 0 & \cdots & 0 \end{array}\right) & \begin{array}{c} \pi_{I}\\ \pi_{I}\\ \cdots \\ \pi_{I} \end{array}\\ \begin{array}{ccccccc} \frac{\delta t}{T_{C}} & \frac{\delta t}{T_{C}} & \cdots & \cdots & \cdots & \cdots & \cdots \end{array} & f_{I} \end{array}) \end{array}$$ (11)

where the cell cycle duration *T*_*C*_ is set to 24*h* [30].

Hence, the deconvolution problem can be understood as a multiple linear regression and, therefore, we can infer the gene expression in the space of the cell-cycle pseudotime by optimizing the following quadratic loss function:

$$\begin{array}{c} L_{SM}=\sum_{g}|r_{g}-ME_{g}{|}^{2}+\lambda \sum_{g\tau }|E_{g,\tau +1}-E_{g,\tau }{|}^{2} \end{array}$$ (12)

where we added a smooth ridge regularization term to be able to solve the linear model and avoid overfitting. Then, the solution is $E_{g}^{*}={\left(Q+\lambda I\right)}^{-1}M^{T}r_{g}$, where *Q* = *M*^*T*^*M* and *I* is the regularization matrix.

Finally, To choose the parameter λ, we calculated the Akaike Information Criterion (AIC) and the Bayesian Information Criterion (BIC) scores in function of λ, as follows [31, 32]:

$$\begin{array}{c} \text{AIC}=N_{E}\cdot \chi^{2}+2N_{G}\cdot D \end{array}$$ (13)

$$\begin{array}{c} \text{BIC}=N_{E}\cdot \chi^{2}+2N_{G}\cdot D\cdot \text{log}\left(N_{E}\right) \end{array}$$ (14)

where *N*_*E*_ is the number of experimental time points, *N*_*G*_ the total number of genes, $\chi^{2}=\sum_{g}|r_{g}-ME_{g}^{*}{|}^{2}$ is the minimum error and *D* is the degree of freedoms that, for a multiple linear regression model with smooth ridge regularization, can be calculated as *D* = Tr(*M*((*Q* + λ*I*)^−1^*M*^*T*^). The BIC score tends to introduce a stronger penalty producing a solution more robust against overfitting therefore we chose λ = 0.79 that minimizes the BIC score (see Fig A in S1 Appendix).

### Visualization of the gene expression through heatmaps

To represent the gene expression as shown in Fig 1A, processed EU-RNA-Seq data at the transcript level from [14] were used. Transcript FPKMs from the same gene were then grouped to obtain gene level EU-RNA-Seq data, and all the genes with a low expression on the asynchronous sample (FPKM < 36) were excluded (see Fig A in S1 Appendix). Then, a Z-score was calculated, correcting each FPKM value by subtracting the mean *μ*_*g*_ and dividing by the standard deviation *σ*_*g*_, both *μ*_*g*_ and *σ*_*g*_ calculated over the corresponding gene. Genes were divided into 5 clusters (the optimum number to obtain significantly different profiles) according to their Z-score over time, by using the *KMeans* tool from *sklearn* python library. The heatmap was represented by using *seaborn* python library, ordering the genes of each cluster according to their norm with respect to the corresponding cluster average expression.

### Inference of transcription factor activities

We developed an ISMARA-like model [15] where the expression of a given gene with respect to the cell-cycle pseudotime can be obtained as a linear combination of time-dependent activities of all TFs that can potentially bind its promoter. First, as proposed in [15], we preprocessed our data as follows: first, to revert the z-score transformation performed above we multiplied the gene expression values *E*_*gτ*_ by the standard deviation *σ*_*g*_ and added the average *μ*_*g*_. Second, in order to calculate the log2 expression for all genes, we add a pseudo-count to the corrected *E*_*gτ*_ values, i.e. for every given time *τ*, we ranked all the values higher than zero and we calculated the 5th percentile *pc*_*τ*_. We then added *pc*_*τ*_ to the corresponding *E*_*gτ*_. After that, we calculated ${\hat{e}}_{g\tau }$, i.e. normalized values of the gene expression at a cell-cycle pseudotime *τ*, as follows:

$$\begin{array}{c} {\hat{e}}_{g\tau }={\text{log}}_{2}\;[10^{6}\cdot \frac{E_{g\tau }}{\sum_{g^{\prime }}E_{g^{\prime }\tau }}] \end{array}$$ (15)

we further normalized the expression of genes across pseudotime and genes resulting in $e_{g\tau }={\hat{e}}_{g\tau }-\langle {\hat{e}}_{g}\rangle -\langle {\hat{e}}_{\tau }\rangle +\langle \langle \hat{e}\rangle \rangle$. Finally, we write the linear model as:

$$\begin{array}{c} e_{g\tau }=\sum_{f}N_{gf}A_{f\tau } \end{array}$$ (16)

where *N*_*gf*_ represents the number of binding sites for the TF *f* on the gene promoter *g*; and, *A*_*fτ*_ is the activity of the TF *f* at a given cell-cycle pseudotime *τ*. To build the matrix **N** we used computational predictions of TFBSs from the database SwissRegulon that combines known TF weight matrices, ChIP-seq data and sequence conservation [17]. TFBSs were associated with a gene promoter if they were located within a window of ±500*bp* around its TSS. The binding site matrix was further normalized to ensure ∑_*g*_ *N*_*gf*_ = 0. Note that the TF activities are then zero mean variables.

Then we used least square fitting to obtain the TF activities. To avoid overfitting we included a ridge regularization penalty. To estimate the weight of the regularization we calculated the Mean Square Error (MSE) for a training and a test datasets and we performed a 80–20 cross-validation. A regularization factor λ = 443 was chosen, corresponding to the minimum of the MSE of the test dataset (see Fig C in S1 Appendix). In addition, we calculated the explained variance (EV) of the model, $EV=\frac{\sum_{g,\tau }{\left(e_{g\tau }-e_{g\tau }^{th}\right)}^{2}}{\sum_{g,\tau }{\left(e_{g\tau }-\mu_{g}\right)}^{2}}$, where $e_{g\tau }^{th}$ is the theoretical expression of the gene *g* at internal cell cycle time *τ*, i.e. calculated using the inferred activity *A*_*fτ*_ and the matrix *N*_*gf*_, and *μ*_*g*_ is the mean among all the values *e*_*gτ*_. We obtained a regularization factor λ = 443, corresponding to the maximum of the EV of the test dataset (see Fig C in S1 Appendix), in accordance with the minimum obtained for the MSE.

Regarding the enhancer analysis, the activities of the motifs have been estimated from the deconvolved expressions of the enhancers provided by Palozola et al. [14] through the ISMARA model (see Eq (16)). The sequences of the enhancer regions have been extracted from the human genome hg19 with bedtools and the TF binding sites have been predicted with Motevo [17]. The EV was calculated to rank the most important transcription factors (see paragraph above).

### Visualization of the TF activities through heatmaps

To represent the TFs activities as shown in Fig 2, the TFs were divided into 3 clusters according to their activity dynamics over *τ*. First, we calculated the standard deviation over time of TF activities as $\sigma_{f}=\sqrt{\sum_{\tau }A_{f\tau }^{2}}$ and classify a TF as high amplitude dynamic if *σ*_*f*_ > 0.07. Second, we sorted TFs according to when their maximum activity peak occurred and defined a TF as mitotic active if the peak appeared before *τ*_mit_. Therefore, TFs were classified as either mitotic active, early-G1 active or non-dynamic. The heatmap in Fig 2 was represented by using *seaborn* python library, ordering the TFs in each cluster by the the first reached maximum over *τ*. To represent the TFs activity as shown in Fig E in S1 Appendix only TFs corresponding to genes belonging to the Gene Ontology (GO) category (Cell Cycle—GO:0007049) were considered.

### Core Regulatory Network

To build the core regulatory network (CRN) we selected the TFs that showed a high degree of explanatory power to reproduce the gene expression dynamics. To do that, we assigned a score for each TF based on its contribution to the explained variance by calculating a *reduced* explained variance $EV_{f}=\frac{{\left(e_{g\tau f}-e_{g\tau f}^{{}^{\prime }th}\right)}^{2}}{{\left(e_{g\tau f}-\mu_{g}\right)}^{2}}$, i.e. the EV as shown in the section Inference of transcription factor activities but removing from the model the corresponding TF *f*. Then, we defined the importance score of a TF by the ratio $\frac{EV_{f}}{EV}$ and we ranked all TFs according to this score taking into account that the smaller is the ratio the higher is the impact of the TF on the explanatory power of the model. The cumulative distribution of importance scores shows that only a small subset of TFs have a large contribution on the explanatory power (see Fig G in S1 Appendix). To select the most relevant TFs, we set a cutoff for the importance score where the cumulative distribution starts to flatten giving us 16 TFs (5% of all TFs). Finally, to build the CRN we used the selected TFs and the potential interactions between them according to the binding site matrix N. The CRN shown in Fig 3 was then generated by using the digraph library in Matlab.

### Genes expression dynamics and bookmarking

To establish which genes are associated to FOXA1, as shown in Fig 4A, we took into account the mitotic ChIP-Seq peaks of FOXA1 from [7]. Then, for every peak, we selected the nearest expressed gene, using as references the corresponding TSS and the average point of the selected peak. So, we obtained a list of expressed genes that we defined the genes bound by FOXA1 during mitosis.

To establish which genes tend to be regulated by TFs with high or low MBF [5], as shown in Fig 4C and 4D, we calculated what we called *mitotic binding score* (MBS) for each gene *g* as an average of MBF scores of the factors that can potentially bind the gene promoter weighted by the number of binding sites, i.e:

$$\begin{array}{c} MBS_{g}=\sum_{f}MBF_{f}*\frac{N_{gf}}{\sum_{f}N_{gf}} \end{array}$$ (17)

where the sum runs over the factors for whcih we know the corresponding MBF score. We then ranked genes according to the MBS, and we removed the ones with MBS = 0. The 10% of the genes with the highest MBS were then considered associated with enriched TFs (“enriched genes”), while the 10% of the genes with the lowest MBS were considered associated with the depleted TFs (“depleted genes”).

To obtain genes enriched or depleted in binding sites, we calculated for each gene promoter the total number of binding sites, i.e. ∑_*f*_ *N*_*gf*_ and then the 10% of the genes with largest number of binding sites and the 10% of the genes with smallest number were considered to calculate average expression profiles as shown in Fig 4E and 4F.

## Supporting information

S1 Appendix Supplementary figures. File containing supplementary figures (Fig A-I).
(PDF)

S1 Table Deconvolved gene expression profiles. Gene expression dynamics with respect to the cell-cycle pseudotime as shown in Fig 1E.
(CSV)

S2 Table Inferred TF activity dynamics. TF activities dynamics estimated with the linear model as shown in Fig 2B.
(CSV)

S3 Table Importance scores of TFs. List of TFs ranked according to their power of explaining the gene expression profiles.
(CSV)

S4 Table Average mitotic activity of TFs. List of TFs ranked according to their average mitotic activity.
(CSV)
