# Supplementary material for: Regulation of transcription reactivation dynamics exiting mitosis
Source: PLoS Comput Biol. 2021 Oct 4;17(10):e1009354. doi: 10.1371/journal.pcbi.1009354 (PMC8516288; doi:10.1371/journal.pcbi.1009354)
Supplement: S1 Appendix — File containing supplementary figures (Fig A-I). (PDF) [file pcbi.1009354.s001.pdf]

S1 Appendix: Regulation of transcription reactivation dynamics exiting mitosis

Sergio Sarnataro[1,†], Andrea Riba[1,†], Nacho Molina[1,*]

[1]Institut de Génétique et de Biologie Moléculaire et Cellulaire (IGBMC)

Université de Strasbourg – CNRS – INSERM, Illkirch, France

[†]Equal contribution

[*]molinan@igbmc.fr

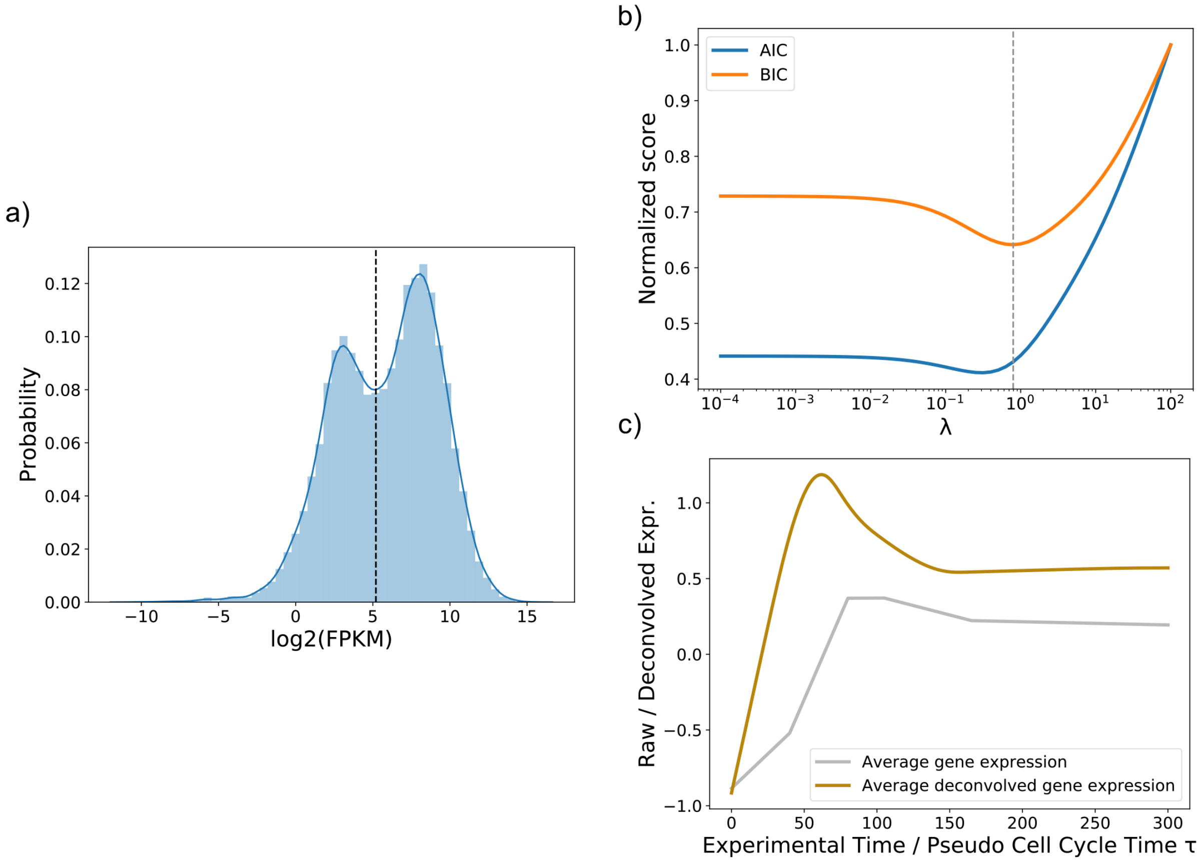

Fig A: Data processing for deconvolution A. Log2 histogram of FPKM reads at gene level for asynchronous data. The dashed vertical line represents the threshold we considered to process our data: genes with asynchronous $FPKM < 36.76$ were excluded. **B**: AIC and BIC scores were calculated in order to establish the best λ parameter for the regularization of the deconvolution process (see Methods in main text). Both AIC and BIC showed a minimum, and we choose $\lambda = 0.79$, corresponding to the BIC minimum (dashed vertical line). **C**: Average gene expression of convolved (grey line) and deconvolved (yellowish line) data were represented on the same plot.

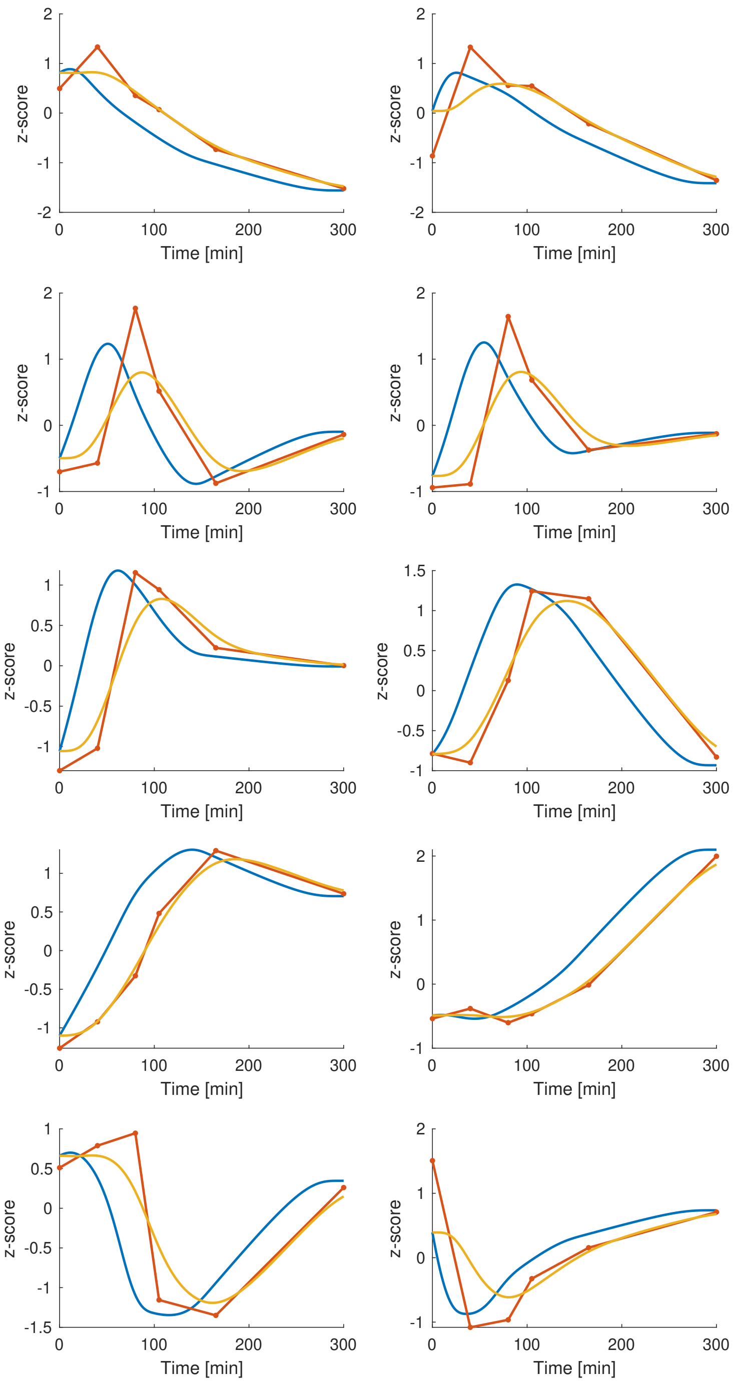

Fig B: Devonvolved gene expression profiles for selected genes. Plots show gene expresion dynamics as measured by Palozola et al. at 6 experimental times after mitotic-blockage release (red dots) together with the decovolved gene expression dynamics after desynchronization is computationally corrected (blue curves). Yellow curves show reconstructed expression profiles applying the convolution operation on the deconvovled gene expression.

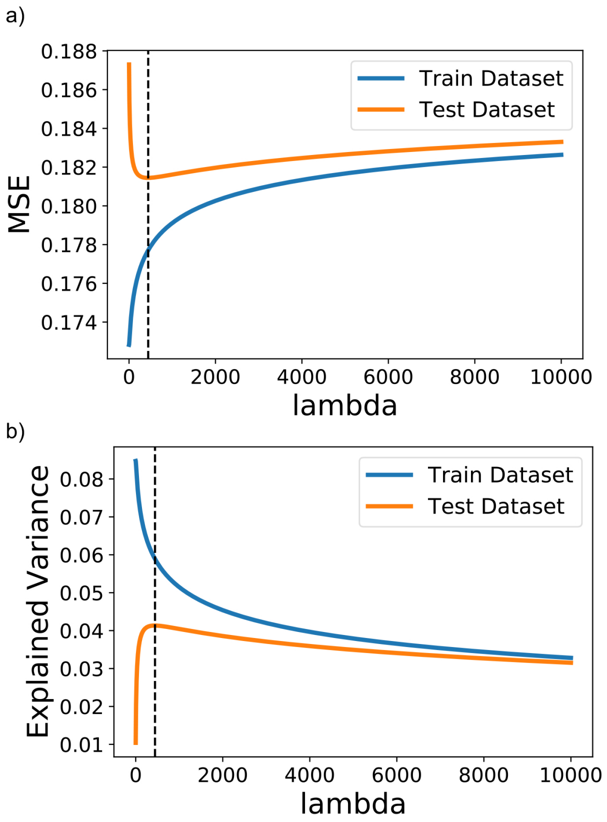

Fig C: Cross validation of the linear model. A. A cross-validation 80/20 was performed to find the best λ regularization parameter for inferring the TFs activity. A value $\lambda = 443$ was chosen (dashed vertical line), corresponding to the minimum of the Mean Squared Error (MSE) of the test dataset (see Methods in main text). **B**: The same analysis shown in the panel A was performed by using Explained Variance (EV) instead of MSE. The dashed vertical line corresponds to the maximum $\lambda = 443$, in accordance with the minimum MSE.

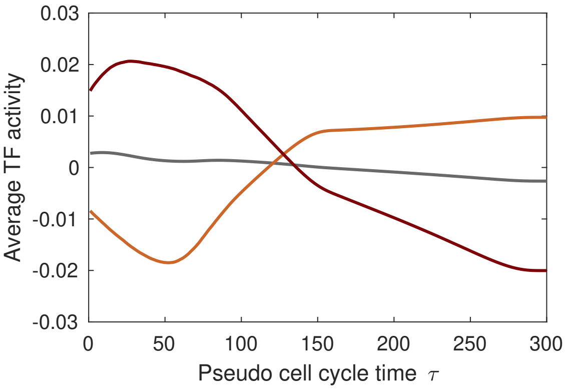

Fig D: Average TF activities in clusters. Cluster mean activities are calculated averaging activities of TFs that belong to the same cluster in Fig 2 in the main text. Three main dynamics are observed: mitotic-active, early-G1-active and steady

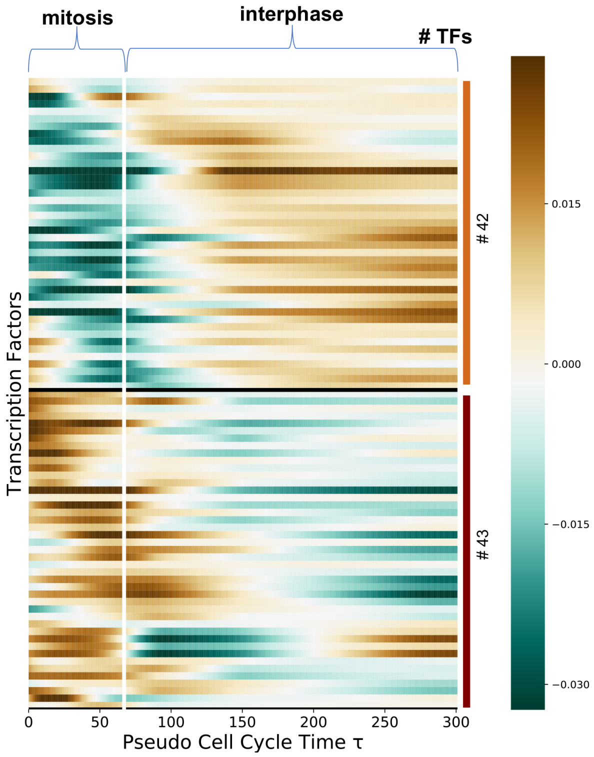

Fig E: Transcription factors dynamics taking into account only cell-cycle GO category. Here, only TFs associated to genes belonging to the Gene Ontology (GO:0007049) category have been shown and clustered. In this case, only 2 main groups of TFs have been individuated, and both of them show a significant activity change over τ. The vertical white line represents τ_{mit}, and separates ideally the mitosis from the interphase. On the right, the number of TFs for every cluster is indicated.

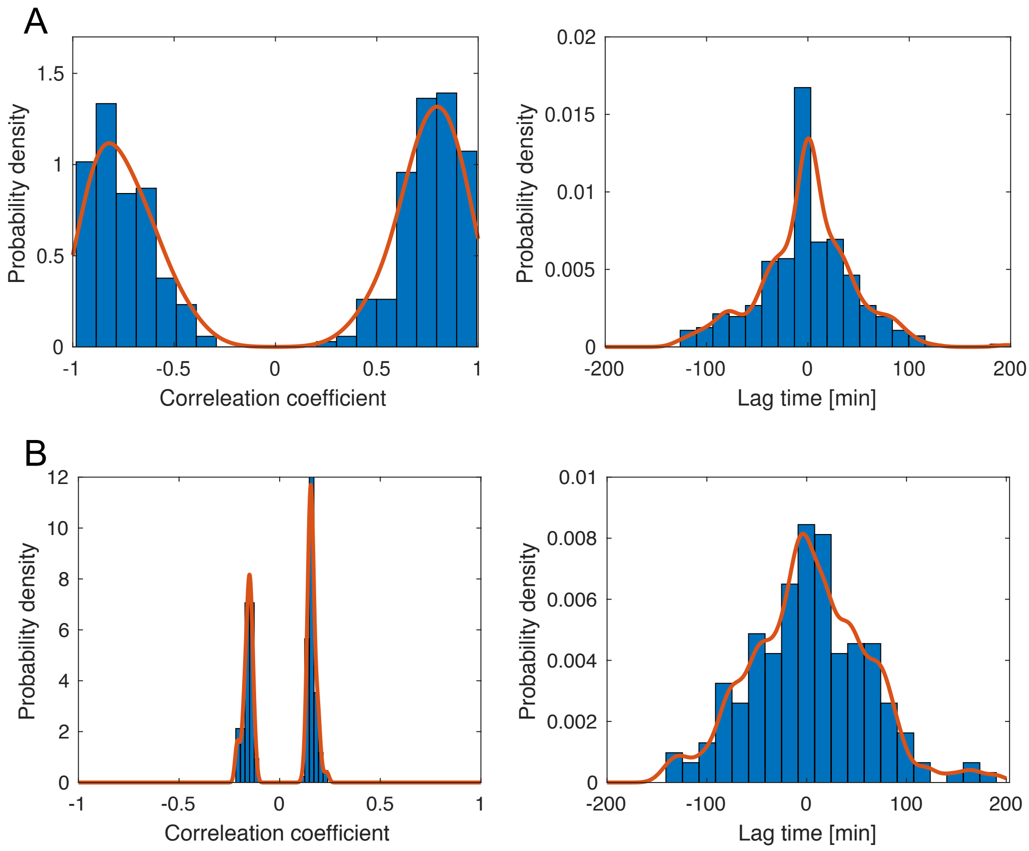

Fig F: Cross-correlation analysis between TF expression and activity. Left: distribution of maximum cross-correlation coefficients. The bimodal distribution may represent TFs that act mainly as repressors (negative cross-correlation) and as activators (positive cross-correlation). Right: distribution of lag times of the TF activity with respect to the TF expression at maximum cross-correlation.

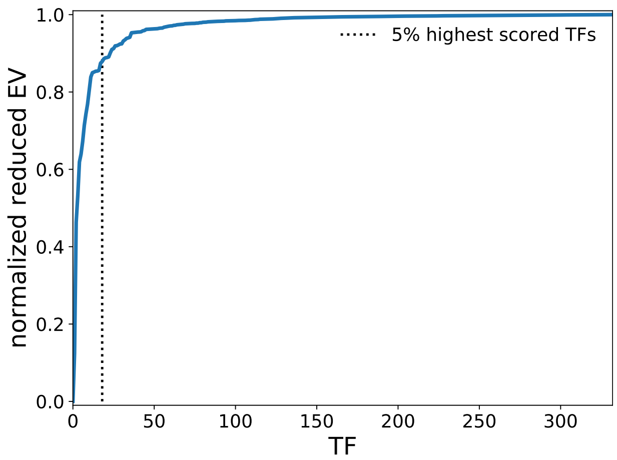

Fig G: Cumulative distribution of reduced explained variance (EV). The curve shows the cumulative distribution of reduced EV values normalized between 0 and 1. Only a small fraction of TFs showed a large reduction in the explained variance when removed from the linear regression. To choose the most relevant TFs for the reactivation of transcription, we set a cutoff where the cumulative distribution starts to flatten which gave us 16 TFs representing 5% of all analyzed TFs. These TFs and the interaction between them according to the binding site matrix N were used to build the CRN in Fig 3 in the main text.

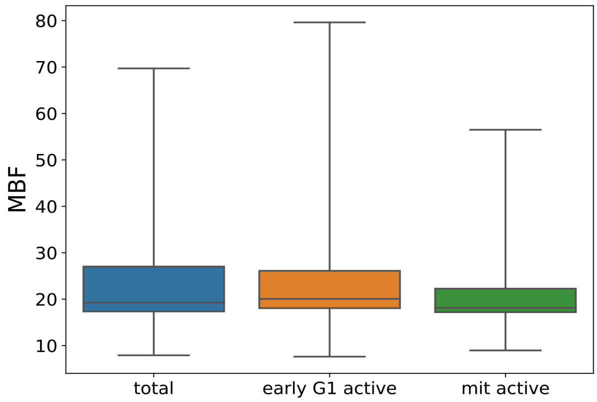

Fig H: Average MBF (Mitotic Bound Fraction) for mitotic and early G1 active transcription factors. Boxplots showing the average MBF for TFs with higher activity during mitotis (green box) and during early G1 (orange box) respectively, in comparison with the average of all TFs (blue box).

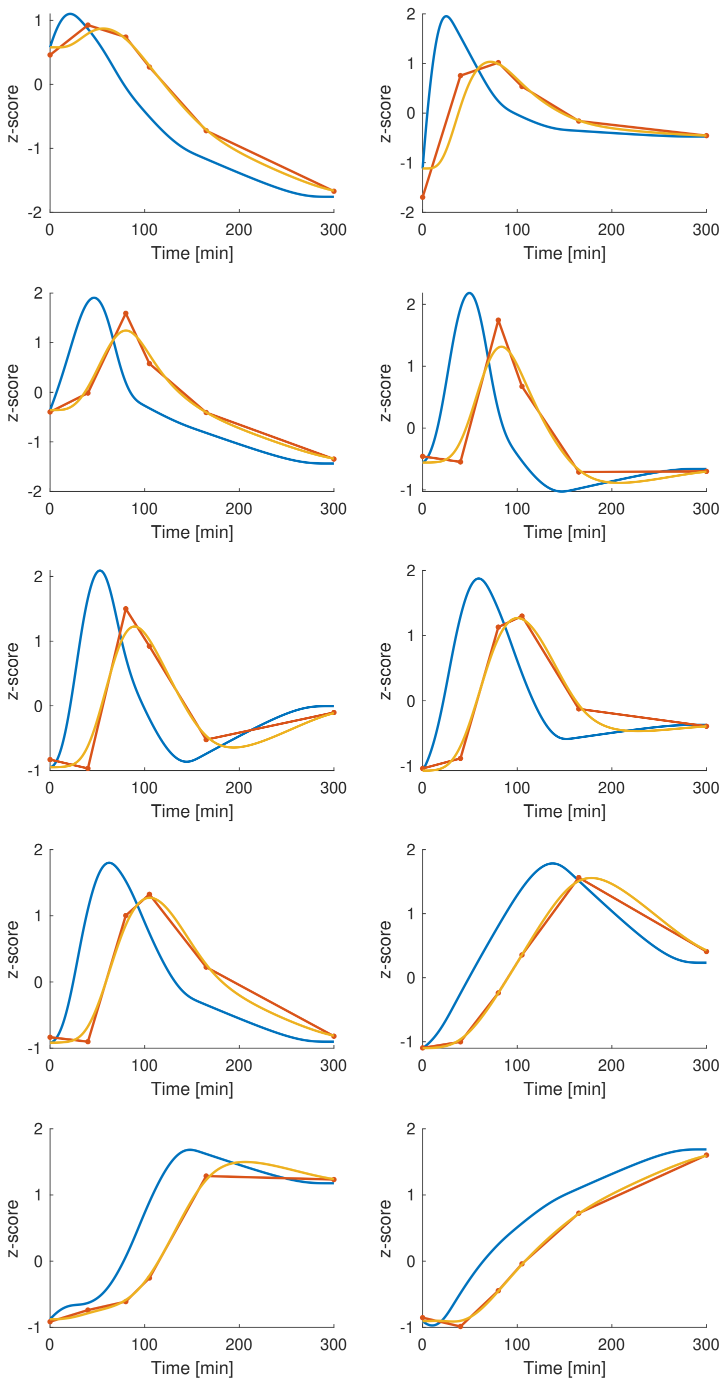

Fig I: Devonvolved eRNA expression profiles for selected enhancers. Plots show eRNA expresion dynamics as measured by Palozola et al. [1] at 6 experimental times after mitotic-blockage release (red dots) together with the decovolved eRNA expression dynamics after desynchronization is computationally corrected (blue curves). Yellow curves show reconstructed expression profiles applying the convolution operation on the deconvovled eRNA expression.
